# Pathogenesis of follicular thymic hyperplasia associated with rheumatoid arthritis

**DOI:** 10.1111/pin.13212

**Published:** 2022-02-11

**Authors:** Rintaro Ohe, Suran Yang, Daisuke Yamashita, Chihiro Ichikawa, Akihisa Saito, Takanobu Kabasawa, Aya Utsunomiya, Naing Ye Aung, Yuka Urano, Takumi Kitaoka, Kazushi Suzuki, Daiichiro Takahara, Akiko Sasaki, Yuya Takakubo, Michiaki Takagi, Mitsunori Yamakawa, Mitsuru Futakuchi

**Affiliations:** ^1^ Department of Pathology, Faculty of Medicine Yamagata University Yamagata Japan; ^2^ Department of Orthopedic Surgery, Faculty of Medicine Yamagata University Yamagata Japan; ^3^ Department of Pathology Kobe City Medical Center General Hospital Kobe Japan; ^4^ Department of Diagnostic Pathology Osaka Women's and Children's Hospital Osaka Japan; ^5^ Department of Diagnostic Pathology National Hospital Organization Kure Medical Center/Chugoku Cancer Center Hiroshima Japan

**Keywords:** follicular helper T‐cell, follicular thymic hyperplasia, germinal center, humoral immunity, memory B‐cell, myasthenia gravis, myoid cell, rheumatoid arthritis

## Abstract

Lymphoproliferative disorders may occur in patients with rheumatoid arthritis (RA) who are treated with methotrexate. However, follicular thymic hyperplasia (FTH) associated with RA (FTH‐RA) is generally not considered a lymphoproliferative disorder. To investigate the pathogenesis of FTH‐RA, we examined 12 cases of FTH involving thymic enlargement, four of FTH involving RA and eight of FTH involving myasthenia gravis (MG). Increased numbers and larger germinal center (GC) size were observed in FTH‐RA group. The percentage of distorted GCs was 13.3% in FTH‐RA group and 3.25% in FTH associated with MG (FTH‐MG) group. A greater meshwork of follicular dendritic cells was observed in the GCs of FTH‐RA group. Positive indices of CD27^+^ cells and PD‐1^+^ cells per GC in FTH‐RA group were significantly higher than those in FTH‐MG group, though positive indices of CD68^+^ cells and CD163^+^ cells were similar. Myoid cell proliferation, as evaluated by α‐SMA, tenascin‐C, and l‐caldesmon expression, was significantly increased in the FTH‐RA group compared with the FTH‐MG group. These results suggest that FTH should be considered in patients with RA treated with methotrexate. The pathogenesis of FTH‐RA includes GC expansion and increased numbers of memory B cells, follicular helper T cells, and myoid cells, indicating humoral immunity activation.

**Abbreviations**
bDMARDs: biological disease‐modifying antirheumatic drugs
CT: computed tomography
EBV: Epstein–Barr virus
EBER‐ISH: Epstein–Barr virus‐encoded small RNA in situ hybridization
FTH: follicular thymic hyperplasia
FDC: follicular dendritic cell
FDG: fluorodeoxyglucose
FTH‐RA: follicular thymic hyperplasia associated with rheumatoid arthritis
FTH‐MG: follicular thymic hyperplasia associated with myasthenia gravis
GC: germinal center
IHC: immunohistochemistry
ISH: in situ hybridization
LPD: lymphoproliferative disorder
MG: myasthenia gravis
MTX: methotrexate
MTX‐LPD: methotrexate‐associated lymphoproliferative disorder
RA: rheumatoid arthritis
RAHA: rheumatoid arthritis hemagglutination
RF: rheumatoid factor
PCR: polymerase chain reaction
RT‐PCR: reverse transcription‐polymerase chain reaction
T_FH_
: follicular helper T‐cell

## INTRODUCTION

The first‐line treatment for patients with rheumatoid arthritis (RA) is methotrexate (MTX). However, treatment with MTX is a risk factor for lymphoproliferative disorders (LPDs) that result from immunosuppression.^1^ Although most MTX‐associated LPDs (MTX‐LPDs) improve after MTX withdrawal, in some cases the clinical course is exacerbated, resulting in aggressive lymphoma.^2^ For patients with RA with poor MTX therapeutic efficacy or those with poor prognostic factors for RA, additional treatment with biological disease‐modifying antirheumatic drugs (bDMARDs), such as etanercept, should be chosen.^3^ Importantly, examination by computed tomography (CT) or X‐ray must be conducted to confirm the absence of malignancy or tuberculosis in patients with RA before treatment with bDMARDs. If enlargement of the thymus is detected by such screening, the possibility of MTX‐LPDs should be considered, with thymoma being the most common tumor of the thymus.

Thymic hyperplasia is classified into two major types: follicular thymic hyperplasia (FTH) and true thymic hyperplasia.^4^ FTH is characterized by the presence of lymphoid follicles with germinal centers (GCs) in the thymus, regardless of its size or weight.^4^ True thymic hyperplasia is characterized by increased weight and normal architecture of the thymus that involves the cortex and the medulla.^4^, ^5^ FTH is known to be associated with myasthenia gravis (MG). To our knowledge, only one Japanese patient with FTH associated with RA has been reported.^6^

In this study, we report four patients with RA with asymptomatic thymomegaly whose thymuses were determined to have FTH. To elucidate the pathogenesis of FTH associated with RA (FTH‐RA), we compared the pathogenesis of FTH‐RA with that of FTH associated with MG (FTH‐MG) because FTH is one of the common complications of MG. In FTH‐MG, myoid cells express acetylcholine receptor. In the thymus, specific antigen presentation is induced in the presence of B cells and follicular helper T cells (T_FH_s) in GCs.^7^ It has been reported that the number of synovial‐infiltrating T_FH_s correlates with disease activity in patients with RA.^8^ Therefore, we focused on myoid cells, memory B cells, T_FH_s, and follicular dendritic cells (FDCs), which play major roles in the formation of GCs, and investigated these cells by immunohistochemistry (IHC).

## MATERIAL AND METHODS

### Patients and tissue specimens

A total of 22 patients were included in this study; the clinical information of 12 of these patients is summarized in Table 1. Twelve FTH samples were obtained from patients with RA (*n* = 4) or MG (*n* = 8). The four patients with RA who participated in this study were diagnosed according to the 2010 American College of Rheumatology (ACR)/European League Against Rheumatism (EULAR) Criteria for RA^9^ and the 1987 American College of Rheumatology Criteria for RA.^10^ To compare the maximum thymus diameter by CT scan, another 10 patients with RA who had not yet received bDMARD treatment were randomly selected from Yamagata University Hospital. Pathological diagnoses were made at Yamagata University Hospital, Kobe City Medical Center General Hospital, and National Hospital Organization Kure Medical Center/Chugoku Cancer Center between 2008 and 2018. Tissues were fixed in 10% neutral‐buffered formalin for 6–12 h at room temperature, embedded in paraffin, and used for hematoxylin‐eosin staining, IHC staining, in situ hybridization (ISH), polymerase chain reaction (PCR), and reverse transcription‐PCR (RT‐PCR). All cases were reviewed by experienced pathologists (OR and KT) and diagnosed according to the Armed Forces Institute of Pathology (AFIP) Atlas^11^ and a previous report.^4^ This study was approved by the Research Ethics Committee of Yamagata University Faculty of Medicine (2018‐253) and was performed in accordance with the Declaration of Helsinki.

**Table 1:** Clinical findings of follicular thymic hyperplasia cases

Case no.	Primary disease	Complication	Age	Sex	Duration	Therapy before resection	RAHA	Anti‐AChR
1	Rheumatoid arthritis	None	40	F	9 years	Methotrexate, Etanercept	×320	NA
2	Rheumatoid arthritis	None	58	F	11 years	Methotrexate	×320	NA
3	Rheumatoid arthritis	None	70	M	NA	NA	NA	NA
4	Rheumatoid arthritis	None	Fifties	F	11 years	PSL, Salazosulfapyridine	×80	NA
5	Myasthenia gravis	B1 thymoma	43	M	3 months	Atorvastatin Ca	NA	40 nmol/L
6	Myasthenia gravis	None	59	F	8 months	Atorvastatin Ca	<×40	1530 nmol/L
7	Myasthenia gravis	B2 thymoma	28	M	NA	None	NA	170 nmol/L
8	Myasthenia gravis	B2 thymoma	38	M	5 months	None	NA	350 nmol/L
9	Myasthenia gravis	B2 thymoma	60	F	3 months	None	NA	82.4 nmol/L
10	Myasthenia gravis	None	30	M	16 months	Pyridostigmine bromide, PSL	NA	36 nmol/L
11	Myasthenia gravis	B2 thymoma	42	F	4 months	Tacrolimus	NA	150 nmol/L
12	Myasthenia gravis	Thymic cyst	66	M	15 years	PSL, Tacrolimus	NA	33 nmol/L

Abbreviations: AChR, anti‐acetylcholine receptor antibody; GC, germinal center; NA, not available; PSL, prednisolone; RAHA, rheumatoid arthritis hemagglutination.

#### IHC

Immunostaining was performed as previously described.^12^ To focus on the composition of cells in GCs and interfollicular/T cell areas, we used antibodies specific for CD20 (L26; mouse IgG2a, κ, DAKO, Agilent Technologies, Santa Clara, California), κ (FLEX Polyclonal Rabbit, DAKO, Agilent Technologies), λ (FLEX Polyclonal Rabbit, DAKO, Agilent Technologies), CD21 (1F8; mouse IgG1, κ, DAKO, Agilent Technologies), CD23 (1F12; mouse IgG1, κ, Novocastra, Leica Biosystems, Nussloch, Germany), D2‐40 (D2‐40; mouse IgG1, κ, DAKO, Agilent Technologies), CD27 (137B4; mouse IgG1, Abcam, Cambridge, UK), PD‐1 (NAT; mouse IgG1, Abcam), CD68 (PG‐M1; mouse IgG1, κ, Novocastra, Leica Biosystems), CD163 (10D6; mouse IgG1, Novocastra, Leica Biosystems), α‐SMA (1A4; mouse IgG2a, κ, DAKO, Agilent Technologies), tenascin‐C (rabbit polyclonal, Epitomics, Abcam, Cambridge, UK), l‐caldesmon (L‐CALD; mouse IgG1, Acris, OriGene Technologies, Rockville, MD, USA), and rheumatoid factor (RF) (005; mouse IgM, GenWay Biotech, San Diego, CA, USA), which reacts to IgM‐RF. A positive reaction when using the labeled streptavidin biotin method resulted in brown coloration with 3,3’‐diaminobenzidine tetrahydrochloride (Dojindo, Kumamoto, Japan).

Immunoreactivity was evaluated as follows. Cell immunopositivity was evaluated in two different ways (area occupied by positive cells/area of the GC [%] and positive cells/total cells in each area [%]) in GCs as well as in the interfollicular/T cell area. Counting was performed in five areas per specimen. Measurements of the size, diameter, square, and positive cell areas by hematoxylin and eosin staining and IHC were performed using HALO software (Indica Labs, Corrale, NM, USA) (Figure S1).

#### Epstein–Barr virus (EBV)‐encoded small RNA ISH (EBER‐ISH)

Paraffin‐embedded thymic tissue sections from patients diagnosed with FTH‐RA (*n* = 4) and FTH‐MG (*n* = 8) were assessed. A paraffin‐embedded colonic lymphoma section (*n* = 1)^13^ was used as a positive control for EBER‐ISH, which was performed with a BOND‐III (Leica Biosystems) Autostainer using BOND EBER Probe (Leica Biosystems).

#### PCR analysis of immunoglobulin heavy chain and T cell receptor gamma

PCR was used to confirm *immunoglobulin heavy chain* and *T cell receptor gamma* gene rearrangement in all patients following the methods described in previous reports.^14^, ^15^

#### RT‐PCR

Paraffin‐embedded thymic tissue sections from patients diagnosed with FTH‐RA (*n* = 4) and FTH‐MG (*n* = 8) were assessed. A paraffin‐embedded synovial tissue section from one patient with RA (*n* = 1) was used as a positive control for RT‐PCR analysis of IgM‐RF mRNA expression because IgM‐RF is known to be produced by synovial B1 cells.^16^ RT‐PCR was performed as previously described.^17^

### Statistical analysis

The Mann–Whitney U test was used to compare the number and diameter of GCs and immunoreactivity. Statistical analyses were performed using JMP, version 14 (SAS Institute, Tokyo, Japan). Differences at *p* < 0.05 were considered significant.

## RESULTS

### Clinical findings

Patient 1: A 40‐year‐old woman who had RA for 11 years had been treated alternatively with tacrolimus/MTX or etanercept. Her serum RA hemagglutination (RAHA) level was 320 times the normal level. Two years prior, a single mediastinal mass was accidentally found by CT scan. Because the mass was considered a tumor, it was removed by thymectomy; at the time, it measured 27 × 20 × 13 mm^3^.

Patient 2: A 58‐year‐old woman who had RA for 11 years had been treated with MTX. Her history included Sjögren's syndrome with unknown onset. Her serum RAHA level was 320 times the normal level. Two years prior, a single mediastinal mass was accidentally found by CT scan. Fluorodeoxyglucose (FDG)‐positron emission tomography revealed accumulation of FDG (SUVmax = 5.4) in this mediastinal mass. The mass, which measured 45 × 30 × 10 mm^3^, was removed by thymectomy.

Patient 3: A man in his 70 s had a history of RA. His history of drug treatment for RA was unknown. A single mass with multinodular lesions in the anterior mediastinum was accidentally detected by CT scan. FDG‐positron emission tomography showed accumulation of FDG (SUVmax = 7.6) in the anterior mediastinum. The mass was removed by thymectomy and measured 65 × 55 × 20 mm^3^.

Patient 4: A woman in her 50s had a history of RA for 10 years and had been treated with prednisolone. Her serum RAHA level was 80 times the normal level. A single mediastinal mass was accidentally found by CT scan prior to valvular surgery for mitral insufficiency. The mass was removed by thymectomy; it measured 30 × 30 × 11 mm^3^.

### Radiological findings of FTH‐RA compared with the thymuses of the patients of RA

**Figure 1:**
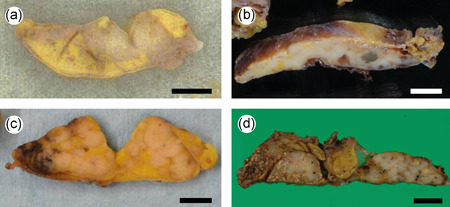
Macroscopic findings of follicular thymic hyperplasia in patients with rheumatoid arthritis. (a–d) There were well‐defined white masses within thymuses (Patients 1‐4). (b–d) A mass is formed by the combination of many nodules (Patients 2–4). a, Patient 1; b, Patient 2; c, Patient 3; d, Patient 4. Bars, 1 mm

Thymus size was evaluated radiologically. The size of the thymus in FTH‐RA group was compared with that in the randomly selected RA group before bDMARD treatment. The maximum thymus diameter in the RA group (FTH‐RA, *n* = 4, Cases 1–4) (Figure 1) was significantly larger than that of the randomly selected RA group before bDMARD treatment (*n* = 10) (41.8 ± 17.4 mm vs. 17.4 ± 3.37 mm, *p* < 0.01).

### Pathological findings of FTH‐RA compared with FTH‐MG

**Figure 2:**
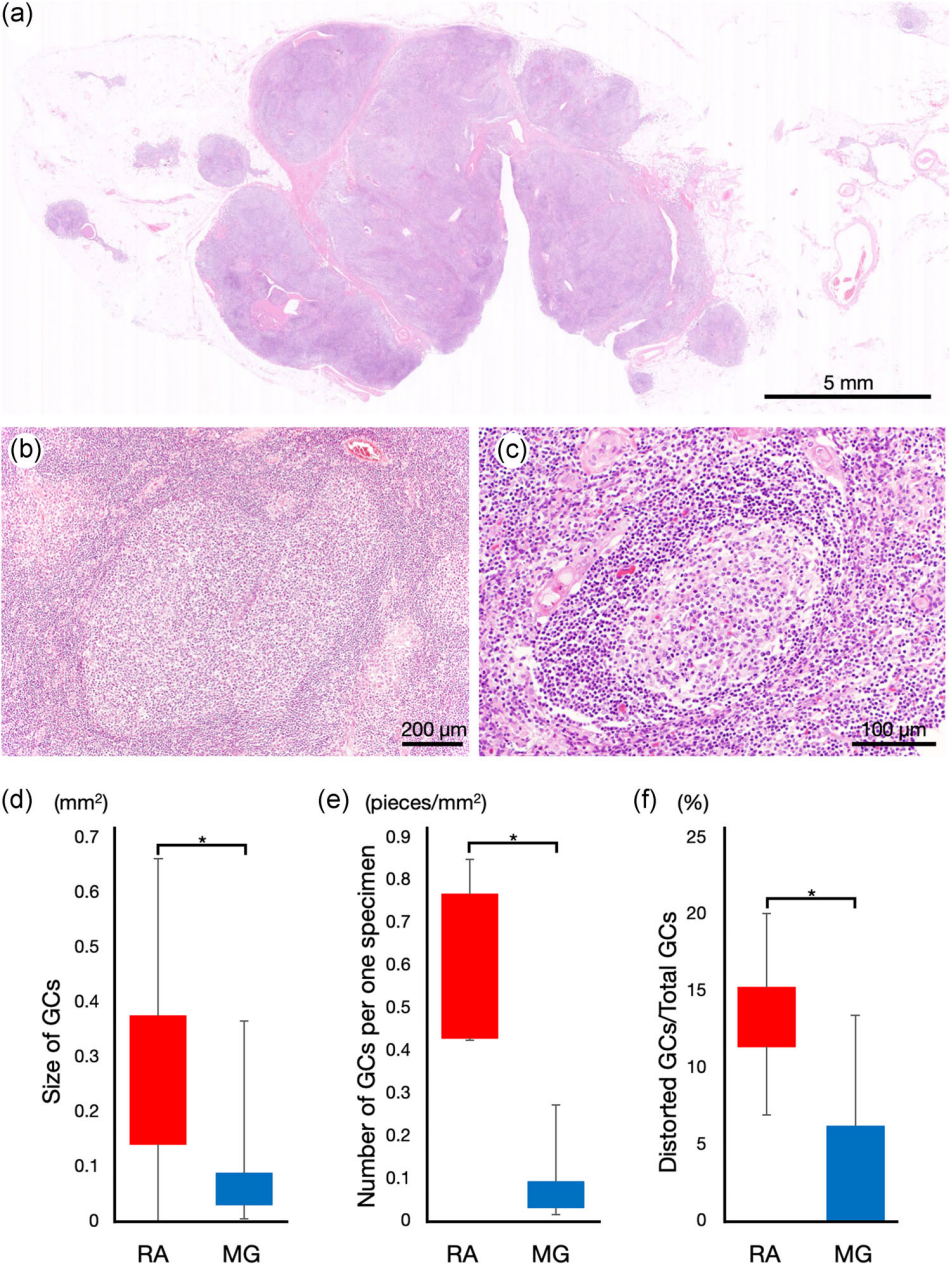
Histological findings of follicular thymic hyperplasia in rheumatoid arthritis (FTH‐RA) and in myasthenia gravis (FTH‐MG). (a) Gross image of an FTH‐RA sample shows numerous lymphocytic infiltrates. (b) The germinal center (GC) in the FTH‐RA sample is large and distorted in shape. (c) The GC of the FTH‐MG sample compared with that of the FTH‐RA sample. (d) The size of GCs in FTH‐RA group was significantly larger than that in FTH‐MG group (FTH‐RA vs. FTH‐MG; 0.33 ± 0.16 mm^2^ vs. 0.073 ± 0.079 mm^2^). (e) The number of GCs in FTH‐RA group was significantly larger than that in FTH‐MG group (FTH‐RA vs. FTH‐MG; 0.61 ± 0.22 pieces/mm^2^ vs. 0.080 ± 0.085 pieces/mm^2^). (f) The average percentage of distorted GCs was 13.3% in FTH‐RA group and 3.25% in FTH‐MG group

In all cases, the thymus showed many secondary lymphoid follicles with GCs (Figure 2a), and these GCs exhibited clear polarity and tingible body macrophages. Moreover, many small, round cells had infiltrated the interfollicular/T cell area, and according to IHC analysis, most were positive for CD20 expression. We then determined that all the thymuses examined displayed FTH but not thymoma according to a previously established criterion.^4^, ^11^ Hematoxylin‐eosin and IHC study revealed that scatter of plasma cells and Ig light chain restriction was not observed in these thymuses. Therefore, we ruled out the possibility of extranodal marginal zone lymphoma of mucosa‐associated lymphoid tissue (MALT lymphoma).

To exclude EBV‐positive LPD (EBV‐positive reactive hyperplasia), we performed an EBER‐ISH study for all patients, with no positive findings indicating EBV infection (data not shown). We also performed PCR to confirm *immunoglobulin heavy chain* and *T cell receptor gamma* rearrangements in FTH‐RA group. But we could not observe any positive findings indicating monoclonality (data not shown).

Furthermore, we compared the pathological findings of FTH‐RA group with those of FTH‐MG group, as summarized in Table 2. The size of GCs in FTH‐RA group was significantly larger than that in FTH‐MG group (Figure 2b–d), and the number of GCs in FTH‐RA group was significantly greater (Figure 2e). Although most GCs were oval shaped, a few were distorted in both groups, with 13.3% in FTH‐RA group and 3.25% in FTH‐MG group (Figure 2f). IHC staining of CD23, which labels the light zone of GCs,^18^ revealed a higher number of GCs with clear polarity in FTH‐RA group.

**Table 2:** Immunohistochemical (IHC) findings of follicular thymic hyperplasia (FTH) associated with rheumatoid arthritis (RA) compared with FTH associated with myasthenia gravis (MG)

Object	Antibody/area	RA (*n* = 4)	MG (*n* = 8)	*p* value
FDC (%, positive area/GC)	CD21/GC	7.76 ± 3.52	2.26 ± 2.20	<0.01
	CD23/GC	15.4 ± 9.19	15.7 ± 8.77	0.7242
	l‐caldesmon/GC	17.8 ± 7.60	9.66 ± 6.47	<0.01
	D2‐40/GC	9.96 ± 8.47	2.88 ± 3.36	<0.01
Memory B‐cell (%, cells/total cells)	CD27/GC	21.6 ± 10.0	13.3 ± 12.5	<0.01
Follicular helper T‐cell (%, cells/total cells)	PD‐1/GC	45.1 ± 18.3	15.1 ± 10.5	<0.01
M1&M2 macrophage (%, cells/total cells)	CD68/GC	11.2 ± 9.07	6.98 ± 3.78	0.2012
M2 macrophage (%, cells/total cells)	CD163/GC	1.05 ± 2.64	2.65 ± 5.87	0.1667
Myoid cell (%, positive area/IF)	α‐SMA/IF	5.92 ± 5.95	0.55 ± 0.49	<0.01
	Tenascin‐C/IF	4.05 ± 3.22	0.53 ± 0.88	<0.01
	l‐caldesmon/IF	12.6 ± 5.89	6.07 ± 5.89	<0.01
IgM‐RF deposition on HC and capillary endothelium	IgM‐RF	100% (4/4)	25% (2/8)	0.0606*

Abbreviations: FDC, follicular dendritic cell; GC, germinal center; HC, Hassall's corpuscle; IF, interfollicular area/T‐cell zone.

*Fisher exact test.

**Figure 3:**
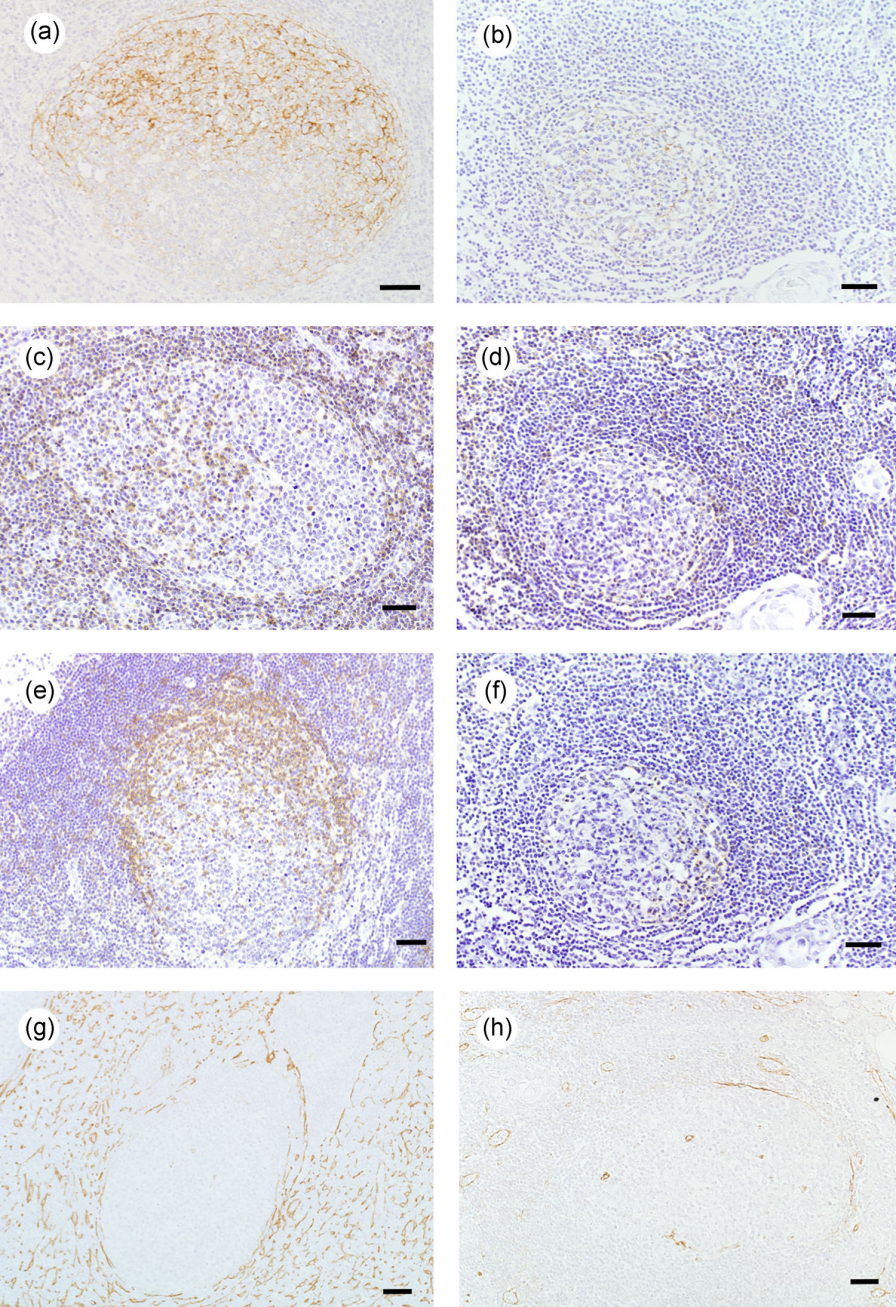
Immunohistochemical findings of follicular thymic hyperplasia in rheumatoid arthritis (FTH‐RA) and in myasthenia gravis (FTH‐MG). (a,b) The CD21^+^ follicular dendritic cell meshwork was thicker and denser in FTH‐RA GCs (a) than in FTH‐MG GCs (b). (c,d) CD27^+^ memory B cells were more abundant in FTH‐RA GCs (c) than in FTH‐MG GCs (D). (e,f) PD‐1^+^ follicular helper T cells were more abundant in FTH‐RA GCs (e) than in FTH‐MG GCs (f). (g,h) α‐SMA^+^ myoid cells/fibroblastic reticular cells were more abundant in FTH‐RA GCs (g) than in FTH‐MG GCs (h). Bars, 50 μm

To investigate the state of humoral immunity activation in GCs, we counted the number of FDCs, memory B cells, T_FH_s, and macrophages. The FDC numbers in GCs were evaluated by using IHC staining for CD21, CD23, l‐caldesmon, and D2‐40. The positive index of CD21 per GC in FTH‐RA group was significantly higher than that in FTH‐MG group (Figure 3a,b); similarly, the positive indices of l‐caldesmon and D2‐40 per GC in FTH‐RA group were significantly higher than those in FTH‐MG group (Table 2). However, there was no significant difference in the positive index of CD23 per GC in between FTH‐RA group and FTH‐MG group. The numbers of memory B cells in GCs were assessed by CD27 staining, the numbers of T_FH_s by PD‐1 staining, and the numbers of macrophages by CD68 and CD163 staining. The positive indices of CD27 per single GC (Figure 3c,d) and of PD‐1 per GC (Figure 3e, f) were significantly higher in FTH‐RA group than in FTH‐MG group (Table 2). Nevertheless, the positive indices of CD68 and CD163 were similar between these two groups (Table 2). IHC for α‐SMA, tenascin‐C, and l‐caldesmon expression was employed to evaluate myoid cell proliferation in the interfollicular/T cell area (i.e., outside the GC), with higher scores in FTH‐RA group (Figure 3g, h; Table 2). IgM‐RF staining was positive in Hassall's corpuscle and the capillary endothelium in all FTH‐RA samples and some FTH‐MG samples (Figure S2; Table 2).

### RT‐PCR of FTH‐RA and FTH‐MG samples

RT‐PCR was performed to detect the presence of IgM‐RF mRNA in FTH‐RA and FTH‐MG samples. Although GAPDH mRNA was detected in all the samples, IgM‐RF mRNA was not found in FTH‐RA or FTH‐MG samples (data not shown).

## DISCUSSION

In this study, we found the thymuses of the RA group (*n* = 4) to be enlarged, with greater maximum diameters than those of the RA group prior to the introduction of bDMARDs (*n* = 10). When an enlarged thymus is found in a patient with RA before the introduction of bDMARDs, histological investigation is recommended to rule out MTX‐LPD.

Hyperplastic GCs were observed in these four thymuses. In general, GCs promote differentiation of B cells into plasma cells and play a central role in humoral immunity activation.^19^ Additional treatment with bDMARDs is required for RA with severe disease progression.^3^ In the present study, severe disease progression was observed in two of the four RA cases (Patient 1 and Patient 2), with both requiring bDMARD treatment. Because hyperplastic GCs were observed in the thymuses of the four patients with RA, we diagnosed FTH. Hyperplastic GCs are often observed in the thymuses of MG patients,^7^, ^11^, ^20^ and it is recommended that these thymuses be removed because thymectomy occasionally improves the clinical outcomes of patients with MG.^20^, ^21^ Our results showed significant GC formation in FTH‐RA group, with greater sizes and numbers than in FTH‐MG group, suggesting that humoral immunity is activated in FTH‐RA group.

GC formation may be observed in synovitis in RA as a result of humoral immunity activation;^16^ it has also been reported that GC formation occurs in the thymus of patients with MG.^7^, ^22^, ^23^ In FTH‐MG, GCs in the thymus produce anti‐acetylcholine receptor antibodies,^23^ and thus GCs in FTH‐MG are associated with humoral immunity activation. Taken together, the results suggest that GC formation is associated with humoral immunity activation in both RA and MG, regardless of thymectomy.

Immunostaining for FDC markers, such as CD21 and CD23, was stained in the FDC cytoplasm in GCs, but the meshwork‐like result made it very difficult to count the exact number of FDCs. In a previous study, we reported 28.1% IHC staining of CD21 in an FDC meshwork, as based on semiquantitative analysis, and we found 10.5% IHC staining of CD23 in tonsils.^18^ According to our previous report,^18^ we evaluated the number of FDCs by semiquantitative analysis of IHC staining of CD21, CD23, l‐caldesmon, and D2‐40 expression. Because FDCs are known to play a crucial role in the regulation of humoral immunity through the long‐term persistence of antibody production,^24^ the results of our study demonstrate activation of humoral immunity, and the number of CD21^+^, l‐caldesmon^+^, and D2‐40^+^ FDCs was significantly increased in FTH‐RA group compared with FTH‐MG group. These results indicate that humoral immunity is activated in the thymus and synovium of patients with RA. Taken together, the results indicate that humoral immunity is activated in the thymuses of patients with severe RA, which might cause thymic enlargement. CD21‐, l‐caldesmon‐, and D2‐40‐positive cells were observed in both the dark zone and light zone of GCs, whereas CD23‐positive cells were observed only in the light zone. Hence, our results suggest enlargement of the dark zone which indicate increasing the number of centroblasts,^25^ but not the light zone. This may be the reason why there was no significant difference in the number of CD23^+^ FDCs between the FTH‐RA and FTH‐MG groups.

Memory B cells and T_FH_s are involved in humoral immunity. Memory B cells are also known to play an important role in long‐term protective humoral immunity.^26^ T_FH_s have important functions in promoting antigen presentation, B‐cell maturation, and antibody production.^7^ All of the roles participate in humoral immunity. Our IHC studies revealed significantly higher numbers of both CD27^+^ memory B cells and PD‐1^+^ T_FH_s in the GCs of FTH‐RA group than FTH‐MG group. We also found that FTH‐RA group had higher humoral immune activity than FTH‐MG group because the number and size of GCs, which are involved in humoral activity, were significantly higher in FTH‐RA group. Therefore, our results indicate that humoral immunity is more highly activated in FTH‐RA than in FTH‐MG. Overall, our histological analysis of humoral activity was consistent with previous studies.^7^, ^26^

FTH‐MG is characterized by ectopic GC development in the thymus,^4^, ^7^, ^23^, ^27^ and thymuses affected by FTH‐MG have a different histological structure from normal thymuses (i.e., thymic remodeling).^27^ Thus, GC development induces thymic remodeling. Because myoid cells are mainly localized in the thymic medulla, GC development may increase the number of myoid cells, and we found that GC number and size were significantly higher in FTH‐RA group than in FTH‐MG group. Hence, GC development in the thymus may be associated with an increased number of myoid cells in FTH‐RA compared with FTH‐MG. However, there is a report that the number of myoid cells differs among patients with MG.^27^ Further studies are necessary to elucidate the mechanism underlying the increase in myoid cells in FTH‐RA compared with FTH‐MG.

We acknowledge that there are several limitations in this study. As we only investigated four patients with FTH‐RA, it is necessary to include more patients in clinical data analysis in future studies. In addition, the study samples were limited to formalin‐fixed paraffin‐embedded tissues, and more studies are needed to elucidate the detailed relationship between FTH‐RA or FTH‐MG and humoral immunity. Because the distorted GC formation may be related to the abnormal highly activation of humoral immunity in our results, more studies are needed to elucidate the detailed the distorted GC formation. Furthermore, thymectomy could be adapted to improve the symptoms of FTH‐RA, as this procedure can sometimes lead to improvements in those with early‐onset MG.

In conclusion, the possibility of FTH should be considered as an LPD in patients with RA who are treated with MTX. The pathogenesis of FTH‐RA involves GC expansion and increased numbers of memory B cells, T_FH_s, and myoid cells, indicating humoral immunity activation.

## CONFLICT OF INTERESTS

All authors declare that there are no conflict of interests.

## AUTHOR'S CONTRIBUTIONS

Rintaro Ohe was responsible for conception and design; Rintaro Ohe, Suran Yang, Daisuke Yamashita, Chihiro Ichikawa, Akihisa Saito, Akiko Sasaki, Yuya Takakubo, and Michiaki Takagi performed the collection and assembly of data; Rintaro Ohe, Takanobu Kabasawa, Aya Utsunomiya, Naing Ye Aung, Yuka Urano, Takumi Kitaoka, Kazushi Suzuki, Daiichiro Takahara, Mitsunori Yamakawa, and Mitsuru Futakuchi performed data analysis and interpretation; Rintaro Ohe, Mitsunori Yamakawa, and Mitsuru Futakuchi wrote the manuscript. All authors have approved the final manuscript.

## Supporting information

Supplementary information.

Supplementary information.

Supplementary information.
