# Supplementary figures and images for: Pathogenesis of follicular thymic hyperplasia associated with rheumatoid arthritis
Source: Pathol Int. 2022 Feb 11;72(4):252–60. doi: 10.1111/pin.13212 (PMC9304286; doi:10.1111/pin.13212)

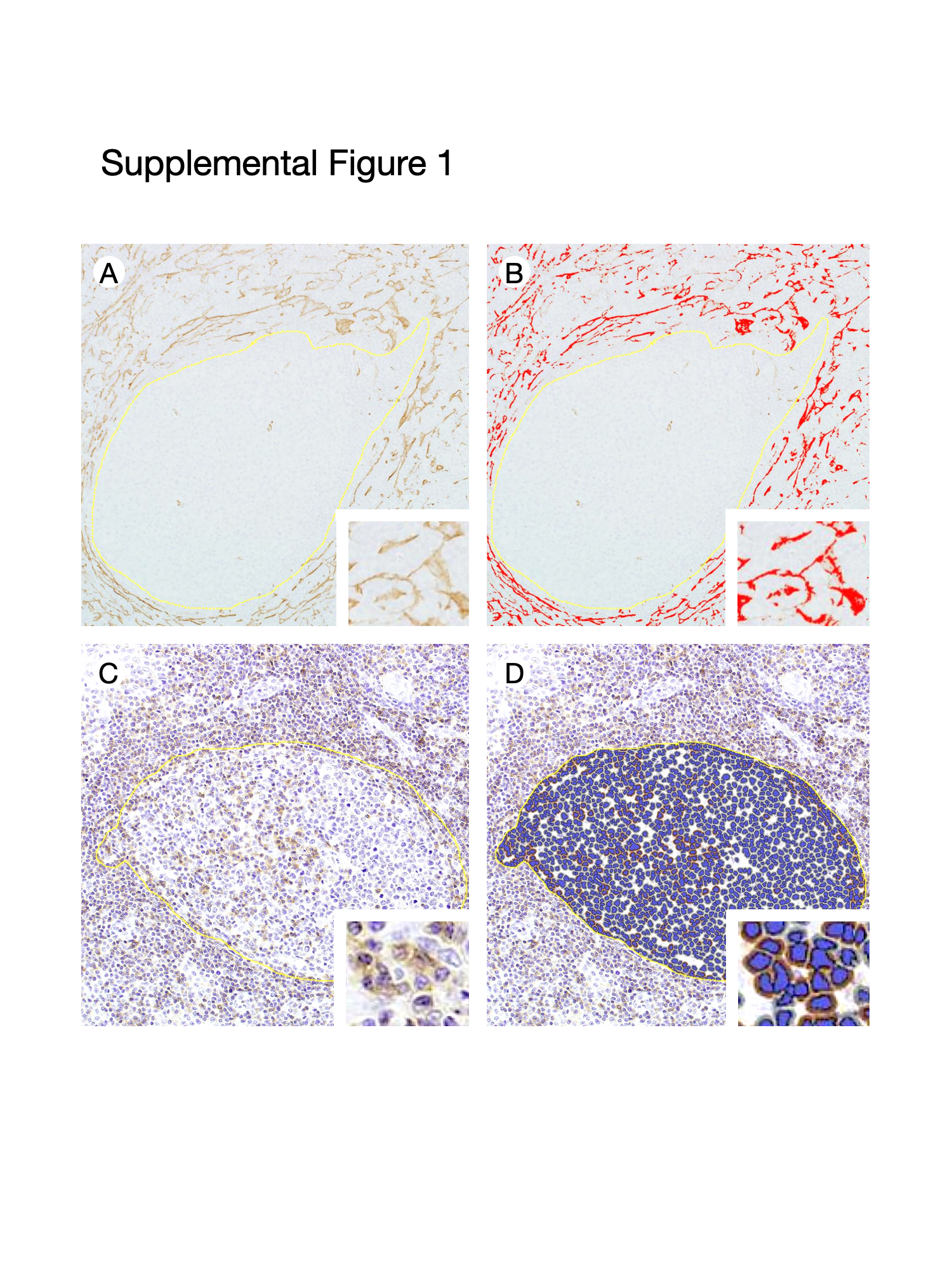

Supplement: Supplementary file 1 — Supplementary information. [file PIN-72-252-s001.tiff]

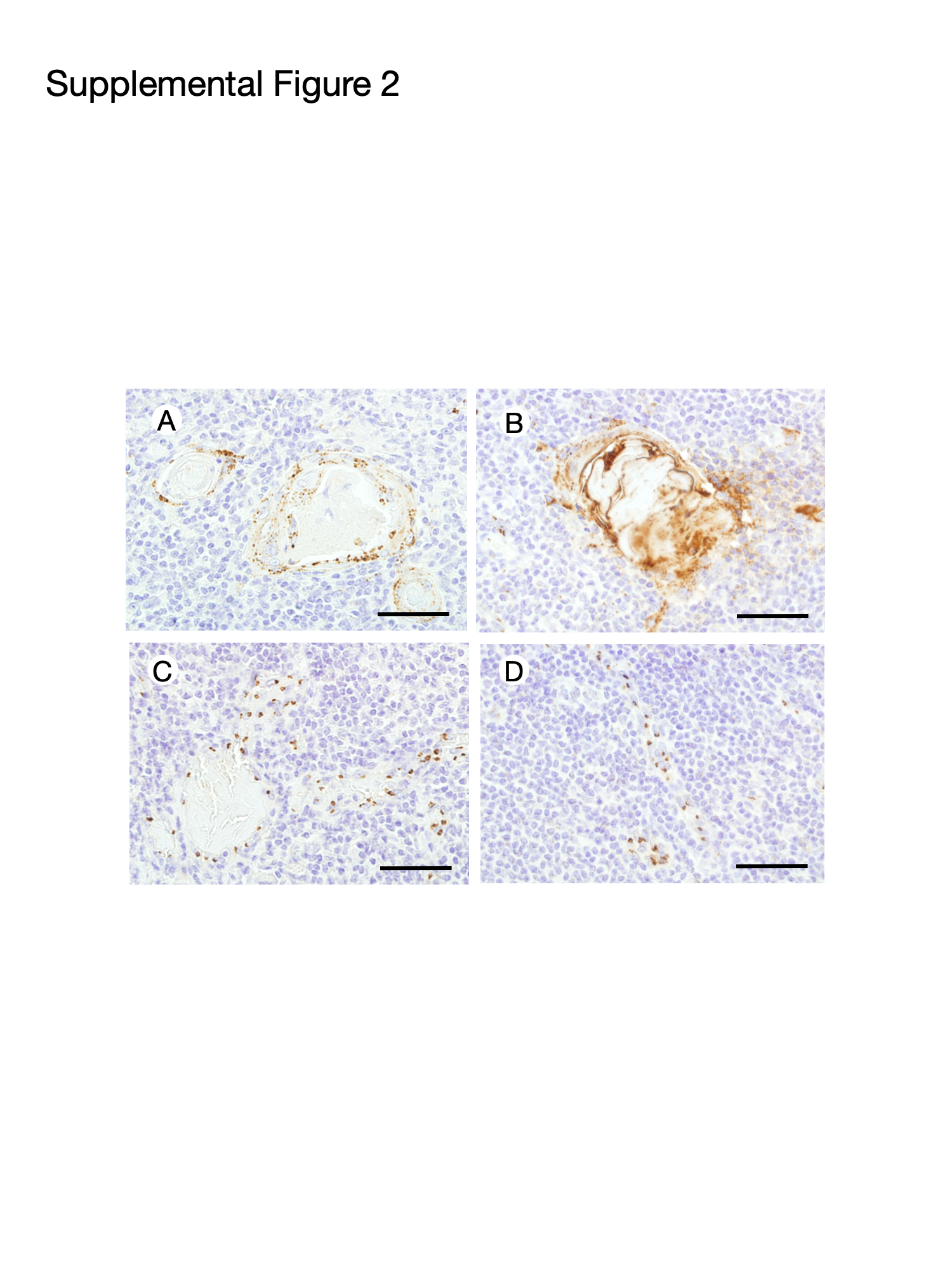

Supplement: Supplementary file 2 — Supplementary information. [file PIN-72-252-s002.tiff]
