# Supplementary material for: Pathogenesis of follicular thymic hyperplasia associated with rheumatoid arthritis
Source: Pathol Int. 2022 Feb 11;72(4):252–60. doi: 10.1111/pin.13212 (PMC9304286; doi:10.1111/pin.13212)
Supplement: Supplementary file 3 — Supplementary information. [file PIN-72-252-s003.docx]

**Supplemental** Figure [1](#MEP_L_fig1)**. Digital image analysis.***(A & B)* Methods of α-SMA-positive cell quantification. α-SMA immunostaining *(A)* and a high-power view of α-SMA-positive cells *(A: Inset)* were labeled red using HALO software (Indica Labs, Corrales, NM, USA) *(B, red: Inset)*. The ratio of the α-SMA-positive area to the background area was evaluated automatically by measuring the α-SMA-positive area *(red)* and background area except the area (germinal center) encircled by the yellow line with the aid of HALO software. *(C & D)* Quantification of CD27-positive cells. Nuclei of CD27-positive cells and other negative cells *[C and Inset (high-power view)]* were labeled dark blue *(D and Inset)*. The plasma membrane of CD27-positive cells was labeled brown *(D and inset)*. The ratio of CD27-positive cells/total cells within a germinal center circled with the yellow line was evaluated with the aid of HALO software.

**Supplemental Figure 2. Immunohistochemical findings of IgM-RF of follicular thymic hyperplasia in the patients with rheumatoid arthritis compared with those with myasthenia gravis.***(A, B)* IgM-RF immunostaining of Hassall's corpuscle was diffusely positive in all four patients with RA *(A)* and in three of eight patients with MG *(B)*. *(C, D)* IgM-RF immunostaining was positive in a paranuclear pattern of small dots in the capillary endothelium in all four patients with RA *(C)* and in three of eight patients with MG *(D)*. Bars, 50 μm.
